# Advancing the women’s health strategy: a policy lab aimed at improving effective management of hyperemesis gravidarum

**DOI:** 10.1186/s12961-025-01384-y

**Published:** 2025-10-09

**Authors:** Melanie Nana, Tianne Haggar, Harriet Boulding, Ross Pow, Catherine Nelson-Piercy, Catherine Williamson

**Affiliations:** 1 Department of Women’s Health, King’s College London, Strand, London, WC2R2LS UK; 2 The Policy Institute, Faculty of Social Science and Public Policy, King’s College London, Strand, London, WC2R2LS UK; 3 Department of Metabolism, Digestion and Reproduction - Faculty of Medicine, Imperial College London, South Kensington Campus, London, SW72AZ UK

**Keywords:** Hyperemesis gravidarum, Women’s health, Policy

## Abstract

**Background:**

Hyperemesis gravidarum (HG) is associated with morbidity and mortality which can be exacerbated by difficulties accessing guideline-recommended treatment. Improving outcomes for women with HG through policy and practice fulfils the vision of the Women’s Health Strategy for England. We aimed to align with the Women’s Health Strategy by addressing barriers to evidence-based care and to improve effective management of HG.

**Methods:**

A Policy Lab was held in Central London in February 2023 guided by the King’s Policy Institute eight-step process for delivery.

**Results:**

The Policy lab brought together 22 key stakeholders from a range of backgrounds in clinical medicine, academia, policy, Royal Colleges and patient representatives. The challenges and opportunities for improving the quality and consistency of care for women with HG were discussed. A long list of 26 possible actions were determined of which five have been implemented and a further six are currently being implemented. Key outcomes include an update on the Royal College of Obstetricians and Gynaecologist (RCOG) guideline on Nausea and Vomiting to enable standardized care across the healthcare settings, awareness of the condition being raised through a number of high impact conferences/educational meetings and HG now being recognized in the Women’s Health Strategy. The Department of Health and Social Care has convened a group focussed on implementing the recommendations of the lab.

**Conclusions:**

This Policy Lab brought together key stakeholders to determine strategies to enhance the Women’s Health Strategy and to ensure all women with HG are able to access guideline-recommended evidence-based treatment. Outputs of the Lab to date include updating the RCOG guideline with the support of Royal Colleges from all relevant care sectors to enable standardized practice.

## Background

Hyperemesis gravidarum (HG) describes debilitating nausea and vomiting of pregnancy which affects up to 3.6% of the pregnant population [1]. Physical complications include weight loss, dehydration, electrolyte imbalance and thrombosis, in addition to morbidity from co-existing conditions in patients unable to take regular time-critical medications (for example corticosteroids for adrenal insufficiency) [2, 3]. The condition also adversely affects mothers’ mental health. In a UK study of > 5000 women we reported that 5% with the condition terminate a wanted pregnancy and 7% experience regular suicidal ideation [4, 5]. Risk factors for these adverse outcomes include difficulty accessing guideline-recommended care [4, 5]. The consequences of poor access to care stretch beyond those experienced by the patients and their family to economic and social costs. The condition represents the commonest cause of hospital admission in the first trimester of pregnancy and costs the NHS up to £62 million per annum as a result of hospital admissions, ambulance call-outs and visits to primary care practitioners [6–8].

Improving outcomes for women with HG through changes to policy and practice fulfils the visions of the 10-year Women’s Health Strategy for England, straddling two priority areas (pregnancy and mental health), and addresses all six priorities for transformational change [9]. Improvement in management requires prompt diagnosis and early access to evidence-based therapies, better health care practitioner information and education and ensuring women are listened to by healthcare professionals.

The adverse consequences of HG can often be avoided through prompt recognition and management of the condition. Best evidence-based practice exists in the form of the Royal College of Obstetricians and Gynaecologists (RCOG) guideline on The Management of Nausea and Vomiting in Pregnancy and hyperemesis gravidarum [10] and European Association for the Study of the Liver (EASL) Clinical Practice Guidelines [11]. However, appropriate implementation of these guideline-recommended treatments is infrequent. Following the tragic death of a pregnant woman by suicide in 2022 a UK parliamentary debate was held to raise awareness of the condition, demonstrating the urgency for change and to make a commitment to improve care for women with HG [12]. Here, we report the outcomes of a King’s Policy Institute Policy Lab aimed at aligning with the Women’s Health Strategy by addressing barriers to evidence-based care with the aim of improving effective management of HG.

## Methods

A Policy Lab was held in Central London in February 2023 guided by the King’s Policy Institute eight-step process for delivery [13]. The process includes:

1. Setting aside time for planning
2. Establishing the need and purpose of the Policy Lab
3. Selecting and inviting participants
4. Synthesizing and communicating the evidence
5. Planning the agenda and facilitation
6. Conducting the Policy Lab
7. Reporting the results
8. Creating and supporting the new coalition

The first six steps of this process are described in the methods of this report and the subsequent two in the results.

**Steps 1–2:** *Setting aside time for planning and establishing the need and purpose of the policy lab*. The proposal to consider holding the policy lab was conceived following discussions with women with lived experience of the condition who raised the question as to why, when national guidance exists for the management of HG, they were unable to access recommended care. Recognizing that this was a question important to patients with the potential to enable more equitable access to appropriate care the authors approached the King’s Policy Institute to start the planning phase. The study team determined an overarching question ‘How can policy and practice ensure that evidence-based guidance of the highest quality is followed by all to improve the treatment of hyperemesis gravidarum?’. It was considered that by answering this question using a policy lab would enable contribution of a number of key stakeholders to explore the proposed problem. In parallel, funding applications were submitted to cover the cost of the Policy Lab.

**Fig. 1:**
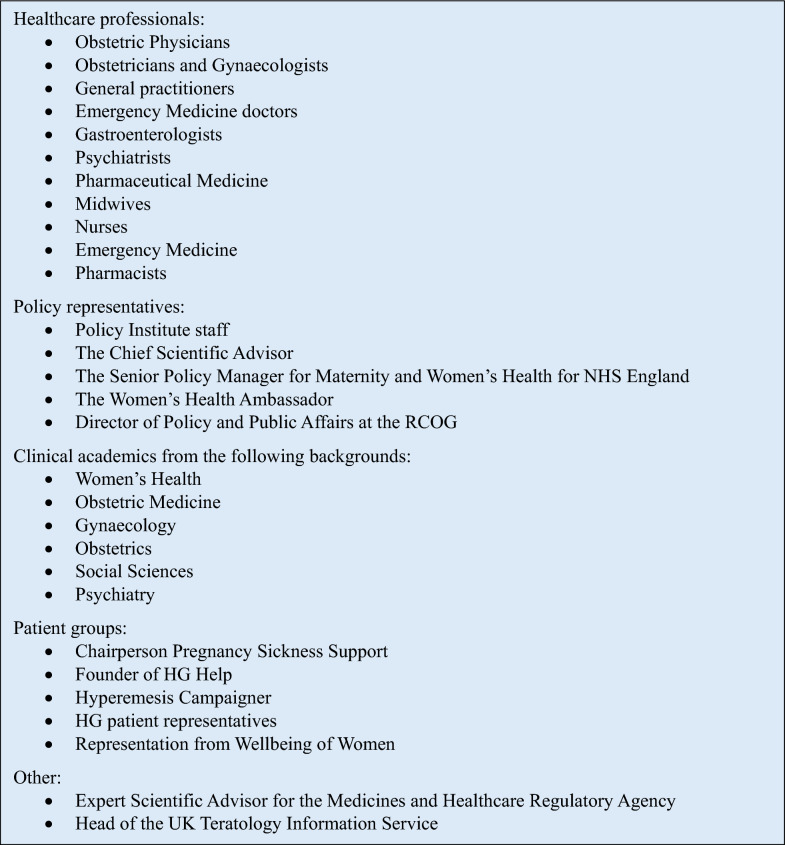
Policy Lab attendees

**Step 3:** *Selecting and inviting participants*. Key stakeholders in the fields of academia, healthcare, the Department of Health and Social Care, NHS England, the Royal Colleges and patient support charities were approached to inform the list of invitees. A priority was to ensure that representation was achieved from all of these areas in addition to ensuring that the representatives were from a variety of gender, ethnic and socioeconomic backgrounds. The final list of attendees is outlined in the results section (Fig. 1). Invitations were sent via email by the senior authors (CW and CNP).

**Step 4:** *Synthesizing and communicating the evidence*. The evidence was collated by the study team (MN, CW and CNP) through review of the relevant literature, discussion with patient representatives and through their experience as clinicians and academics. A briefing pack was assembled to outline the evidence to inform discussion. This covered the following:

*The Impact of HG on health and wellbeing:* The physical and mental health complications of the disease (outlined in the introduction) were summarized, in addition to the adverse fetal and child health outcomes of babies born to women with severe HG [14].

*A summary of the guideline-recommended treatment:* Focussed on the evidence-based guideline developed by the Royal College of Obstetricians and Gynaecologists.

*Potential barriers to treatment*: including:

1. Prescribing anxiety: a number of factors may contribute to the reticence to prescribe in pregnancy and particularly prescribing antiemetic therapies. The 1960s thalidomide (a drug specifically marketed to treat nausea and vomiting of pregnancy) disaster resulted in a reappraisal of all medication use in pregnancy with a fear of teratogenicity. Despite significant changes in drug safety testing and large Cochrane reviews now reporting reassuring safety data regarding the currently recommended antiemetics, confidence in prescribing guideline-recommended treatment is low [15, 16].
2. Knowledge of healthcare professionals: In 2021 we conducted a survey of general practitioners which confirmed poor knowledge and confidence in prescribing in pregnancy of guideline recommended HG treatments. While 93% of respondents identified cyclizine as being safe in pregnancy, no other drug recommended in the RCOG guideline was correctly identified as being compatible with pregnancy by more than 58% of participants [16]. One in five participants did not believe that thiamine (used to prevent development of Wernicke’s encephalopathy) is safe in pregnancy [16]. It is our experience that this lack of knowledge is not confined to the primary care setting. An important finding of the study was that 87% of respondents expressed a desire for better signposting to up-to-date and evidence-base guidance. Notably the most commonly used guideline used in primary care does not reflect existing evidence [17].
3. Attitude of healthcare professionals to women suffering with HG: the attitude towards patients with this condition is reported to be unkind, dismissive and cruel [4, 5]. Reports of women being made to feel as if they are wasting health care professionals (HCPs) time, are unable to cope, or are risking the health of their unborn baby if they seek care is not uncommon.
4. Mixed messaging and variation in national guidance: current guidance for the management of HG has historically not been standardized with significant variation in recommendations between UK national guidance [17, 18].

The briefing pack was distributed via email prior to the policy lab event, made available in paper copy on arrival at the meeting and presented by MN at the beginning of the day. Efforts were made to ensure that it was based on current literature and suitable for an audience of varying literacy levels.

**Step 5–6:** *Planning the agenda, facilitation and conducting the policy lab*: the Policy Lab was held at the King’s Policy Institute, London. The travel costs of all participants were reimbursed to widen access. The structure of the day was based on recommendations from the Policy Institute with introduction of the briefing pack followed by a series of break-out sessions in groups of 5–8 people. A facilitator with expertize in strategic planning and policy development was present, and used his experience to encourage an open environment where participants felt comfortable and able to share their view points. Discussions from the lab followed Chatham House Rules and were captured in several ways including a publicly available output (Supplementary document 1).

## Results

**Steps 7–8:** *Reporting the results and creating and supporting the new coalition:* The Policy lab brought together 22 key stakeholders from a range of backgrounds in clinical medicine, academia, policy, Royal Colleges and patient representatives (some with overlapping roles) (Fig. 1).

The challenges and opportunities for improving the quality and consistency of care for women suffering with HG were discussed and included:

1. *Women with HG should have holistic and individualized support*
  It was recognized that women suffering with HG deserve effective and holistic support so that they can function and carry out their day-to-day activities and minimize the risk of complications of untreated disease. They are not seeking attention by attending the Emergency Department. It is critical that they should feel safe and listened to, their suffering validated, particularly during their initial interactions with healthcare professionals. Holistic individualized support facilitates should facilitate access for women from different backgrounds and circumstances.
2. *The diagnosis and management of HG is multi-faceted*
  The management of HG can be complex often requiring parallel inputs from professionals across primary and secondary services which sometimes results in lack of ownership of the support to women. This, combined with the lack of knowledge about the condition by healthcare professionals across different sectors, results in women feeling misunderstood.
3. *Large parts of the workforce lack sufficient knowledge of HG*
  There are large gaps in the education of HCPs who manage HG patients, the condition is frequently missed from educational curricula resulting in poor awareness of the consequences of untreated disease for both the mother and the offspring. Some current guidelines are outdated and are not consistent across healthcare sectors. More widespread awareness and education around the condition through more inclusive curricula and professional educational training was recognized to have the potential to improve the care of women presenting to different healthcare settings.
4. *The condition has not been prioritized in research, quality improvement (QI) or policy*
  Because nausea and vomiting are so common in pregnancy, affecting up to 80% of women, HG is often trivialized. Lack of investment in both the science to enhance an understanding of the disease aetiology and to evaluate potential treatments, in addition to lack of quality improvement efforts to implement already known best-practice, has resulted in the condition being ‘left behind’ other medical conditions. Prescribing anxiety due to fears of using antiemetics in pregnancy, the public stigma around the condition and it being a ‘woman’s problem’ were proposed as additional reasons for these conditions not being prioritized in research, QI or policy. It was recognized that more investment is needed in the science of HG.
5. *Linking HG into the Women’s Health Strategy and local Integrated Care Board (ICB) plans could raise awareness and drive improvement*
  In 2022 the government detailed an ambitious 10-year strategy to improve the health and wellbeing of women and girls in England. We acknowledged that this offered the opportunity to raise the profile of HG and that any communication from the Policy Lab should reference and mirror the language of the strategy to ensure it resonates with policy makers. It was felt that the opportunity to have HG recognized and explicitly mentioned in national policy could be assisted by aligning with the single delivery plan for maternity and neonatal care. At a local level, it was suggested that promoting better HG care should be encouraged within integrated care boards (ICBs) services and workforce plans. The latter are designed to enable improved care for complex conditions such as HG that transect primary and secondary care and benefit from a multidisciplinary approach.
6. *Conflicting and out-of-date guidelines are barriers to prompt diagnosis and effective management*
  It was strongly agreed that the range of current non-standardized guidelines is resulting in delays in diagnosis and non-evidence-based management. Creating a single set of guidelines across different professional groups was considered an important opportunity for ensuring all women access evidence based best practice.
7. *Examples of good practice should be shared*
  There are examples of novel models being used in some hospitals to improve access for women with HG, sharing of good practice has the potential to enable access to better care for more women in a time efficient manner.

The Policy Lab identified a ‘long list’ of possible actions assessed in terms of their potential impact and feasibility, summarized in Table 1.

**Table 1:** ‘Long list’ of possible actions of which those highlighted in bold have been achieved since the Policy Lab and those in italic are work in active progress at the time of publication

Higher impact/harder to deliver *Raising HG awareness amongst the general population, policy makers, government and healthcare decision makers (for example, within ICBs)* More funding for research and implementation (for example, into effective holistic management, ethnicity data, economic analysis to support HG care, an so on) More GPs with special interest in HG (including working with same day emergency care and ambulatory care settings) Midwife clinical champions Changes to sick/fit line policies More occupational health support *Prioritizing HG management on RCGP, RCOG, midwife, EPU nurse curriculum (for example, CPD for GPs and midwives)* Financial incentives for GPs to manage HG *Development of integrated primary care management package*	Higher impact/Easier to deliver **Developing a single set of guidelines with user-friendly appendices for specific clinical groups and patients** Updating CKS guidelines with supporting clinical practice tools Implementing an MDT approach to the care of HG patients **Tying HG into the Women’s Health strategy** ‘Piggy backing’ on current menopause and other campaigns (for example, oncology) Prescribing powers and related training for midwives and nurses *Shifting the message to the risks of untreated HG on the mother and baby* *Building knowledge amongst women (for example, through health apps, social media, community platforms, popular culture, an so on)*
Lower impact/Harder to deliver *Engaging the wider medical community* Targeting community pharmacy and upskilling over the counter access Extending window for employment appeals against unfair dismissal **Education for pharmacists** Financial incentives for GPs to manage HG	Lower impact/Easier to achieve Home IV fluids Advocacy (from celebrities, patient stories) **Promoting safety of anti-emetics in later pregnancy** **Political pressure (for example, debates, questions in parliaments, an so on)**

*HG* hyperemesis gravidarum; *ICBs* integrated care boards; *GP* general practitioner; *RCOG* Royal College of General Practitioners; *RCOG* Royal College of Obstetricians and Gynaecologists; *EPU* early pregnancy unit; *CPD* continued professional development; *CKS* clinical knowledge summary; *MDT* multidisciplinary team; *IV* intravenous

### Outputs of the policy lab to date

In February 2024 CNP lead the update of the RCOG guideline on Nausea and Vomiting in Pregnancy [10]. MN was a co-author of the guideline and worked with CNP to design appendices with flow charts for evidence-based management for different care settings. The Royal Colleges supported these appendices which have been co-badged by the GPs Championing Perinatal Care (GPCPC), the Association of Early Pregnancy Units, the Royal College of Emergency Medicine and supported by the Royal College of General Practitioners. An example of one of these flow charts can be found in Supplementary Document 2. CW has subsequently led the development of the European Guideline for the Study of the Liver Clinical Practice Guideline which has been co-authored by MN; the section on HG has guidance which reflects that of the RCOG guideline [19]. This guideline has aimed to enable standardized care across the healthcare settings. The authors have now approached the National Institute for Health and Care Excellence NICE with agreement to update the Clinical Knowledge Summary to be in keeping with the RCOG guideline.

Awareness of the condition has been raised through inclusion of sessions on the topic at a number of high impact conferences/educational meetings including the International Society of Obstetric Medicine, the Association of Early Pregnancy Units annual conference, RCOG World Congress, the Fetal Medicine Foundation World Congress, a Royal College of Emergency Medicine national study day, a primary care Guidelines Live conference, the Malaysian Obstetric Medicine meeting amongst a number of regional and local meetings. The Women’s Health Ambassador hosted an online webinar in collaboration with Wellbeing of Women to raise awareness of the condition for women and the authors have recorded a series of podcasts (The Obs Pod) for HCPs.

The Department of Health and Social Care convened a group focussed on implementing recommendations of the Policy lab and to determine research priorities, and HG has now been included in the Women’s Health Strategy. CW is currently working with the RCOG to influence future curriculum development with increased inclusion of HG. The Lancet have subsequently commissioned a review article on HG which is now in press. The authors continue to work with charities to ensure that women’s voices remain at the centre of the work and they have been awarded a subsequent Policy Support Fund grant by King’s College London to carry out health economics work in this area and to further raise awareness and reconvene the key stakeholders at a follow-up event in June 2025. They wish to thank all of the stakeholders who attended the Policy Lab and who have supported implementation of the guidelines, and most importantly the women who continue to share their stories, participate in research and inspire their further work.

### Strengths and limitations

The Policy Lab brought together key stakeholders from a range of backgrounds who worked collaboratively with the support of an experienced facilitator resulting in rich discussion. Inclusion of patient representatives and charities ensured women’s voices remained central to discussions. Engagement of all key stakeholders beyond the time frame of the lab has enabled recommendations to be implemented in a timely fashion.

Policy Labs are considered to rely on ‘political patronage’ [20] and while significant steps were made in engaging political parties in supporting the improvement in HG care, the focus was lost somewhat after the change of government in July 2024. This consolidated the importance of having had HG acknowledged in the Women’s Health Strategy which has remained a focus for the Labour government. Additionally, Policy Labs do not extend to providing a framework for delivery of recommendations which can lose focus without significant determination by the team. The cost of the Lab can be prohibitive; the authors thank the Bikkja Trust for funding this event.

## Conclusions

HG is a debilitating condition which, when left untreated, can result in significant physical and mental health morbidity in women who suffer with it. This Policy Lab brought together key stakeholders to determine strategies to enhance the Women’s Health Strategy by determining strategies to ensure all women with HG are able to access guideline-recommended evidence-based treatment. Outputs of the Lab to date include updating the RCOG guideline with the support of Royal Colleges from all relevant care sectors to enable standardized practice. Future work will include continuing to raise awareness of the condition, continued lobbying for funding to enhance the scientific understanding of the condition and to inform novel treatments and continued efforts to ensure that women’s voices are central to informing policy in this area.

## Data Availability

No datasets were generated or analysed during the current study.

## Abbreviations

HG: Hyperemesis Gravidarum
RCOG: Royal College of Obstetricians and Gynaecologist
EASL: European Association for the Study of the Liver
UK: United Kingdom
HCPs: Health Care Professionals
ICBs: Integrated Care Boards
GPCPC: GPs Championing Perinatal Care
NICE: National Institute for Health and Care Excellence
